# Dissection of the molecular circuitry controlling virulence in *Francisella tularensis*

**DOI:** 10.1101/gad.303701.117

**Published:** 2017-08-01

**Authors:** Bonnie J. Cuthbert, Wilma Ross, Amy E. Rohlfing, Simon L. Dove, Richard L. Gourse, Richard G. Brennan, Maria A. Schumacher

**Affiliations:** 1 Department of Biochemistry, Duke University School of Medicine, Durham, North Carolina 27710, USA;; 2 Department of Bacteriology, University of Wisconsin-Madison, Madison, Wisconsin 53706, USA;; 3 Division of Infectious Diseases, Boston Children's Hospital, Harvard Medical School, Boston, Massachusetts 02115, USA

**Keywords:** *Francisella tularensis*, PigR, MglA, SspA, bioterrorism, ppGpp, virulence

## Abstract

*Francisella tularensis,* the etiological agent of tularemia, is one of the most infectious bacteria known. Because of its extreme pathogenicity, *F. tularensis* is classified as a category A bioweapon by the US government. *F. tularensis* virulence stems from genes encoded on the *Francisella* pathogenicity island (FPI). An unusual set of *Francisella* regulators—the heteromeric macrophage growth locus protein A (MglA)–stringent starvation protein A (SspA) complex and the DNA-binding protein pathogenicity island gene regulator (PigR)—activates FPI transcription and thus is essential for virulence. Intriguingly, the second messenger, guanosine–tetraphosphate (ppGpp), which is produced during infection, is also involved in coordinating *Francisella* virulence; however, its role has been unclear. Here we identify MglA–SspA as a novel ppGpp-binding complex and describe structures of apo- and ppGpp-bound MglA–SspA. We demonstrate that MglA–SspA, which binds RNA polymerase (RNAP), also interacts with the C-terminal domain of PigR, thus anchoring the (MglA–SspA)–RNAP complex to the FPI promoter. Furthermore, we show that MglA–SspA must be bound to ppGpp to mediate high-affinity interactions with PigR. Thus, these studies unveil a novel pathway different from those described previously for regulation of transcription by ppGpp. The data also indicate that *F. tularensis* pathogenesis is controlled by a highly interconnected molecular circuitry in which the virulence machinery directly senses infection via a small molecule stress signal.

## Abstract (precis)

Cuthbert et al. identify MglA–SspA as a novel ppGpp-binding complex and describe structures of apo- and ppGpp-bound MglA–SspA.

Bacteria have evolved multiple strategies to grow within particular hosts. In contrast to opportunistic pathogens, *Francisella tularensis*, the causative agent of tularemia, can infect a wide range of healthy hosts, including mammals, arthropods, and protozoa (Barker and Klose 2007). Enhancing its ability to spread, *F. tularensis* can survive for extended periods in diverse environmental niches. Multiple organisms such as flies, ticks, and mosquitoes also serve as vectors of tularemia transmission to humans (Barker and Klose 2007). The ability of *F. tularensi*s to respond and adapt to diverse stresses has resulted in one of the most infectious agents known. Its pathogenicity along with its ease of dissemination has resulted in its classification as a category A bioterrorism agent. Therefore, specific and potent *Francisella* anti-virulence chemotherapeutics are urgently needed.

The factors and molecular mechanisms that control pathogenicity in *F. tularensis* have a novel biology and are not fully understood. However, a cluster of genes called the *Francisella* pathogenicity island (FPI) has been shown to be essential for *F. tularensis* virulence (Nano et al. 2004; Weiss et al. 2007; Barker et al. 2009; Broms et al. 2010; Dai et al. 2011; Eshraghi et al. 2016). The FPI cluster, which appears to encode an atypical type VI secretion system, is present in two copies in the most virulent *F. tularensis* strains, subspecies *tularensis* and *holarctica* (Broms et al. 2010; Russell et al. 2014; Eshraghi et al. 2016), and its gene products are required for phagosomal escape by the bacteria into the cytosol, an essential step in *Francisella* replication and infectivity (Baron and Nano 1998).

Unraveling the details of FPI regulation has been difficult, as few recognizable transcription regulators are encoded in the *Francisella* genome. Indeed, the transcription system that mediates FPI activation was shown to be composed of an unusual set of regulators: the 210-residue stringent starvation protein A (SspA), the 205-residue macrophage growth locus protein A (MglA), and a 111-residue protein called the pathogenicity island gene regulator (PigR), also known as the *Francisella* effector of virulence regulator (FevR) (Lauriano et al. 2004; Charity et al. 2007, 2009; Brotcke and Monack 2008). SspA proteins belong to the glutathione-S-transferase (GST) family, which contains a thioredoxin fold connected to a helical domain (Oakley 2011; Wu and Dong 2012). SspA proteins, however, do not bind glutathionine (GSH) and are not functional GSTs. In other bacteria, SspA proteins form homodimers, and several of these proteins have been implicated in virulence, including the SspA proteins from enterohemorrhagic *Escherichia coli* (EHEC) (Williams et al. 1994; Hansen et al. 2005; Hansen and Jin 2012), *Neisseria gonorrhoeae* (DeReuse and Taha 1997), *Vibrio cholerae* (Merrell et al. 2002; Xu et al. 2003), and *Yersinia enterocolitica* (Badger et al. 2000). The *F. tularensis* SspA protein, which shares ∼30% sequence identify with other bacterial SspAs, is not homodimeric and instead forms a complex with MglA (Charity et al. 2007; Wrench et al. 2013a; Cuthbert et al. 2015). Interestingly, recent data showed that MglA also harbors a GST-like fold, although this protein shows little sequence homology with SspA proteins (Cuthbert et al. 2015); the *F. tularensis* MglA and SspA share only 19% sequence identity.

Previous experiments suggest that MglA and SspA form a heterodimeric complex (Charity et al. 2007; Cuthbert et al. 2015), making it currently the only known heteromeric SspA complex. Multiple studies have shown that SspA proteins impart their function through interaction with RNA polymerase (RNAP), and, in *Francisella*, both MglA and SspA are required for RNAP interaction (Reeh et al. 1976; Ishihama and Saitoh 1979; Charity et al. 2007). In addition to the MglA–SspA complex, activation of the FPI requires the PigR protein (Charity et al. 2007, 2009). Interestingly, bridge-hybrid assays showed that PigR binds the MglA–SspA complex but does not bind either MglA or SspA alone (Charity et al. 2009). Furthermore, ChIP-seq (chromatin immunoprecipitation [ChIP] combined with high-throughput sequencing) studies demonstrated that PigR colocalizes with MglA–SspA and that all three proteins are required to activate the FPI as well as at least 90 additional genes, some of which have been implicated in virulence enhancement (Charity et al. 2007, 2009; Faron et al. 2013). How PigR collaborates with MglA and SspA to mediate FPI gene regulation is unknown. However, PigR contains a helix–turn–helix (HTH) motif that places it in the MerR family of proteins (Brown et al. 2003). In addition, recent studies raised the possibility that PigR may interact with a 7-base-pair (bp) DNA sequence, named the PigR recognition element (PRE) (Ramsey et al. 2015).

While data have clearly demonstrated that SspA, MglA, and PigR are essential for *Francisella* virulence, how these proteins sense the remarkably wide range of environmental stresses that drive pathogenicity has been a central question in the field. A possible link between infectivity and *Francisella* virulence activation was revealed by studies that demonstrated that FPI induction requires production of the “second messenger,” guanosine–tetraphosphate (ppGpp, used here to refer to both ppGpp and its precursor, pppGpp) (Charity et al. 2009). We now know that this unusual nucleotide functions as a general stress signal in multiple bacteria (Potrykus and Cashel 2008; Hauryliuk et al. 2015; Steinchen and Bange 2016). Enzymes that synthesize ppGpp (RelA/SpoT homologs, referred to as RSH enzymes) are almost universally conserved among all bacteria (Atkinson et al. 2011). In most proteobacteria, including *E. coli*, the primary target of ppGpp is RNAP, and ppGpp regulates hundreds of promoters (Durfee et al. 2008; Traxler et al. 2008) either positively or negatively, depending on the initiation kinetics of the specific promoter (Haugen et al. 2008). Most transcription effects are attributable to direct binding of ppGpp to RNAP together with the RNAP-binding transcription factor DksA (Ross et al. 2013, 2016; Zuo et al. 2013). However, in *Bacillus subtilis* and many other bacterial species outside the proteobacteria, ppGpp does not bind RNAP but rather binds to enzymes responsible for GTP synthesis, inhibiting rRNA and some other promoters that start with and require high concentrations of GTP. Thus, promoter-specific effects on transcription in these cases result from a reduction in the GTP concentration (Krasny and Gourse 2004; Liu et al. 2015). In *Francisella*, DNA microarray studies showed that the global gene expression profiles of Δ*mglA* or Δ*relA*Δ*spoT* mutant strains affected regulation of some of the same genes (Charity et al. 2009), further implicating ppGpp as a molecular signal that helps integrate the stress response of host cell invasion with activation of *Francisella* pathogenicity genes.

To define the molecular determinants that control *Francisella* pathogenicity, we carried out a battery of structural, biochemical, and cellular studies on the key *Francisella* virulence factors. Strikingly, our structural work buttressed by our solution, and in vivo studies revealed that the MglA–SspA heterodimer forms a novel ppGpp-binding complex. Furthermore, we demonstrated that PigR binds with high affinity to MglA–SspA when the heterodimer is bound to ppGpp. Thus, these studies reveal a direct link between the stress-sensing molecule ppGpp and activation of the pathogenicity machinery of *Francisella* via the (MglA–SspA)–(ppGpp)–PigR complex.

## Results

### Structure of the *F. tularensis* MglA–SspA complex

**Figure 1.**
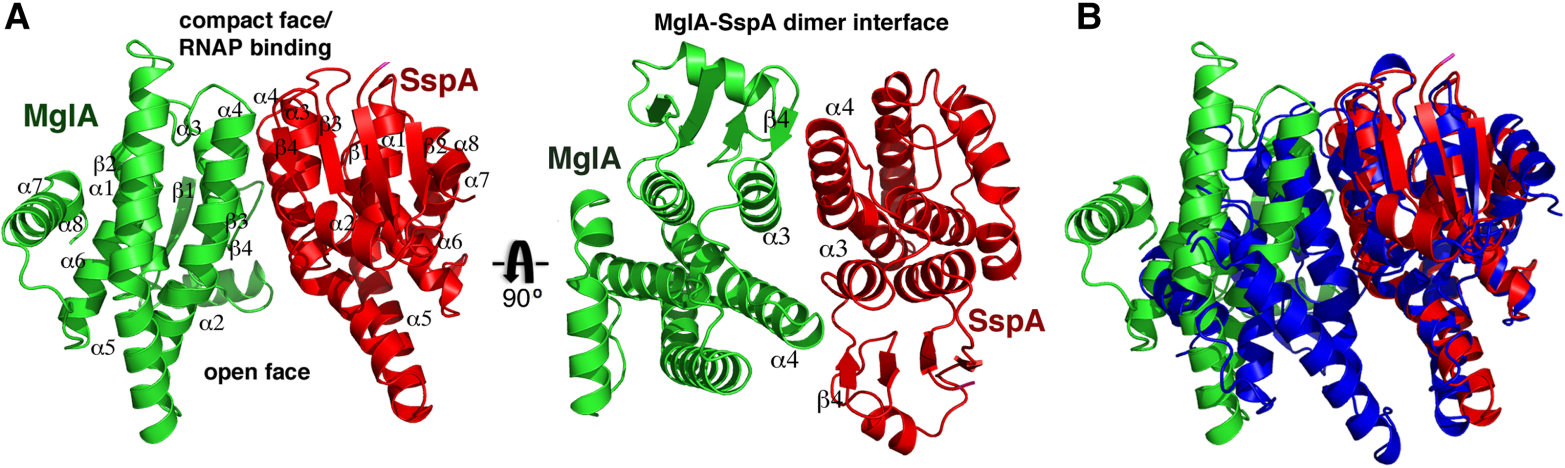
Structure of the MglA–SspA heterodimer. (*A*) The heterodimer is shown as a cartoon, with MglA colored green, SspA colored red, and secondary structural elements labeled. (*Right*) Structure rotated by 90° to better visualize the regions of contact between MglA and SspA. (*B*) Superimposition of the SspA subunit from the MglA–SspA heterodimer (green and red) onto a GST homodimer subunit of structure 1AOF (blue). The overlay shows that MglA–SspA has a more open dimer face on one side.

Previous data have shown that the *Francisella* SspA protein is not stable when expressed alone and requires MglA to be soluble (Wrench et al. 2013a; Cuthbert et al. 2015). In contrast, MglA can be readily expressed as a soluble protein and can form homodimers at high concentrations, although it preferentially interacts with SspA to form the physiologically active MglA–SspA complex that mediates FPI regulation (Charity et al. 2007; Cuthbert et al. 2015). To gain insight into why *Francisella* SspA forms a hetero-oligomer with MglA and the molecular architecture of the complex, we solved and refined the structure of MglA–SspA to final *R*_work_/*R*_free_ values of 20.5%/25.9%, respectively, to 2.65 Å resolution (Table 1; Materials and Methods). As suggested by previous biochemical data, the structure revealed that SspA and MglA combine to form a heterodimeric complex (Fig. 1A; Cuthbert et al. 2015). The structure contains two independent but nearly identical MglA–SspA dimers in the asymmetric unit (ASU). The MglA and SspA subunits contain N-terminal thioredoxin folds followed by a helical domain and show structural similarity to GST proteins (root mean squared deviations [RMSDs] of 1.75–1.84 Å comparing MglA or SspA with GST proteins). However, the MglA–SspA dimer contains an oligomeric architecture distinct from canonical GST proteins, in which one of its “faces” is much more solvent-exposed (Fig. 1A,B). In particular, while the region of the MglA–SspA dimer that is predicted to bind RNAP (Hansen et al. 2005) forms a closed junction, the opposite face is splayed open, creating a large cavity between the MglA and SspA subunits (Fig. 1A,B).

**Table 1.**
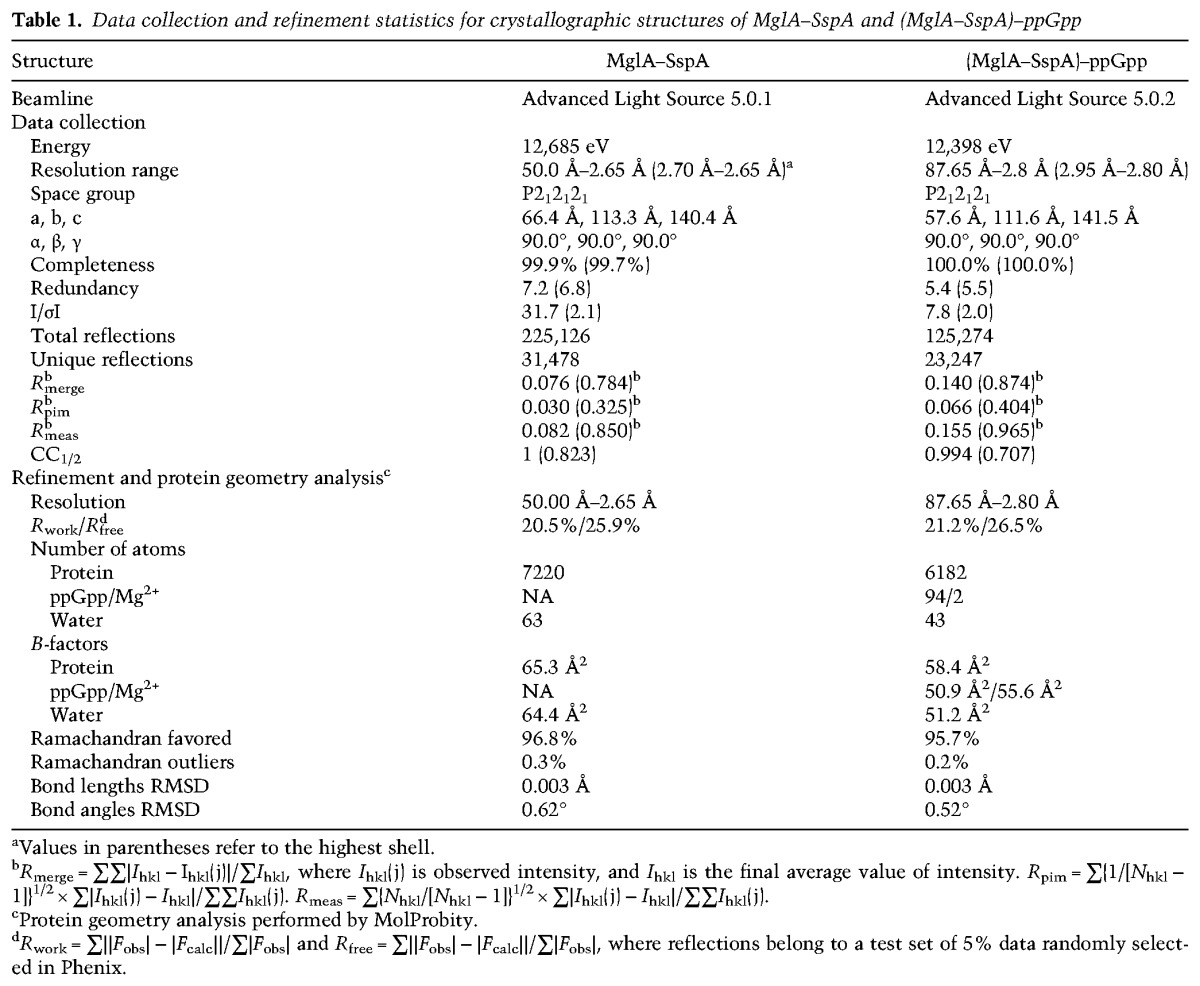
Data collection and refinement statistics for crystallographic structures of MglA–SspA and (MglA–SspA)–ppGpp

**Figure 2.**
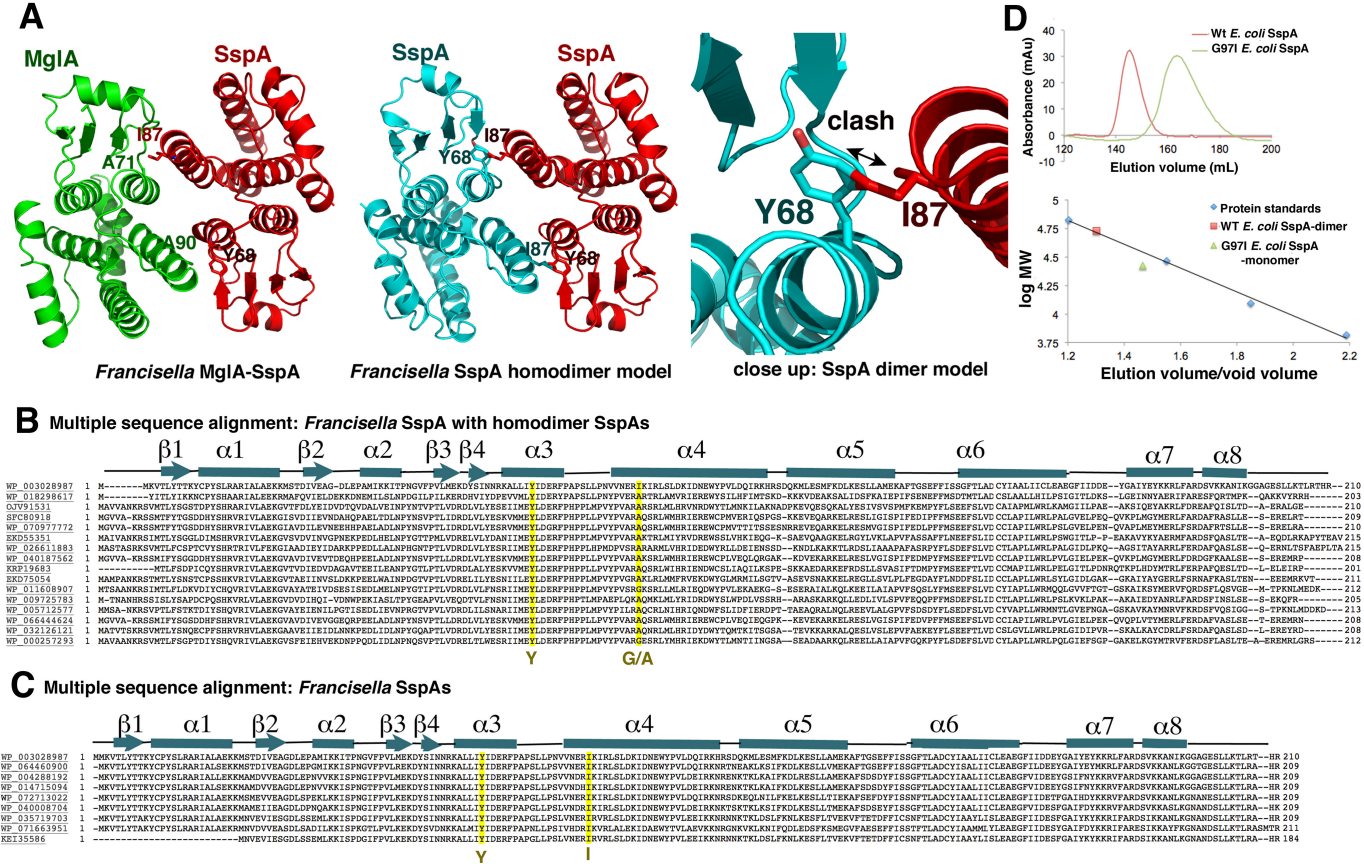
Basis for MglA–SspA heterodimerization. (*A*) Comparison of the MglA–SspA dimer (*left*) with a *Francisella* SspA homodimer model. (*Middle* and *right* panels) Shown are residues Y68 and I87 that would clash in a *F. tularensis* SspA homodimer. This clash does not occur in known SspA homodimers, as the isoleucine is either an alanine or glycine. (*B*) Multiple sequence alignment comparing the *F. tularensis* SspA sequence with non-*Francisella* SspA proteins expected or known to homodimerize: WP_003028987, *F. tularensis* SspA; WP_018298617, *Fangia hongkongensis* SspA; OJV91531, γ-proteobacteria bacterium 39-13 SspA; SFC80918, *Kushneria avicenniae* SspA; WP_070977772, *Kushneria sp*. YCWA18 SspA; EKD55351, uncultured bacterium SspA; WP_026611883, *Methylocaldum szegediense* SspA; WP_040187562*, Halomonas salina* SspA; KRP19683, SAR92 bacterium BACL16 MAG-120619-bin48 SspA; EKD75054, uncultured bacterium (groundwater metagenome) SspA; WP_011608907, *Histophilus somni* SspA; WP_009725783*, Methylophaga lonarensis* SspA; WP_005712577, *Haemophilus parasuis* SspA; WP_066444624, *Halomonas chromatireducens* SspA; WP_032126121, *Piscirickettsia salmonis* SspA; and WP_000257293, *E. coli* SspA. The yellow-highlighted sequences mark the positions of tyrosine and glycine or alanine residues at positions 68 and 90, respectively. (*C*) Multiple sequence alignment comparing the *F. tularensis* SspA protein sequence with other *Francisella* SspA proteins not expected to homodimerize: WP_003028987, *F. tularensis* SspA; WP_064460900, *F. persica* SspA; WP_004288192, *F. philomiragia* SspA; WP_014715094, *F. noatunensis* SspA; WP_072713022, *Francisella sp. TX077310* SspA; WP_040008704, *Francisella sp.* FSC1006 SspA; WP_035719703, *Francisella sp.* W12-1067 SspA; WP_071663951, *Francisella sp.* CA97-1460 SspA; and KEI35586, *Francisella sp*. W12-1067 SspA. The yellow-highlighted sequences mark the positions of residues Y68 and I87. (*D*) SEC analysis of wild-type *E. coli* SspA and the *E. coli* SspA(G97I) mutant. Wild-type SspA (red square) and SspA(G97I) (green triangle) elute at volumes that correlate with molecular weights (MWs) of 51.3 and 34.7 kDa, respectively, indicating that wild-type SspA is dimeric (calculated MW = 52.9 kDa) and SspA(G97I) is monomeric (calculated MW = 26.5 kDa).

Although the MglA–SspA heterodimer is less compact than GST dimers, its dimer interface is still extensive, with a buried surface area (BSA) of ∼1880 Å^2^. This interface is created by the interaction of residues located on loops between β4 and α3 and residues on helices α3 and α4 of both proteins (Fig. 1A). What distinguishes the *Francisella* heterodimer from SspA homodimers and why this bacterium uses this complex are key questions, in particular because of the central role of MglA–SspA in *Francisella* virulence. To examine why *Francisella* SspA does not homodimerize, we constructed a *Francisella* SspA homodimer model (Fig. 2A). Strikingly, this modeling exercise revealed that the side chains of Tyr68 and Ile87 in an *F. tularensis* SspA homodimer would clash, even accounting for different rotamer conformations of the side chains (Fig. 2A). In the MglA–SspA heterodimer, this clash is avoided because the Tyr68 and Ile87 side chains are replaced by alanines in MglA (Ala71 and Ala90, respectively). This finding led us to examine the identities of these residues in homodimeric SspA proteins (Hansen et al. 2005). Sequence alignments of these SspA proteins revealed that while the tyrosine corresponding to *Francisella* SspA Tyr68 is conserved, the residue corresponding to Ile87 is either an alanine or glycine, which allows dimer formation without clash (Fig. 2B). Analyses of SspA homodimer structures (Hansen et al. 2005) confirmed the juxtaposition of the conserved tyrosine with the alanine or glycine residues within the dimer interface. In contrast, sequence alignments of *Francisella* species SspA proteins that form heterodimers with MglA reveal the complete conservation of the tyrosine and isoleucine pair (Fig. 2C) and that their MglA partner proteins contain alanine residues at both of these positions.

MglA prefers to heterodimerize with SspA but can homodimerize at very high concentrations such as used for its crystallization (Cuthbert et al. 2015). Comparison of the crystallographic MglA dimer with the MglA–SspA heterodimer shows that the latter provides favorable charge–charge and polar contacts and complementary hydrophobic interfaces, whereas in the MglA homodimer, there are potential electrostatic clashes. For instance, in the MglA–SspA heterodimer, SspA residue Glu99 makes favorable contacts with MglA residues Tyr63 and Arg64, while in the MglA homodimer, residue Glu99 is replaced by a lysine (Lys101), which does not interact with residue Tyr63 and is proximal to another basic residue, Arg64.

These structural analyses indicate that the *Francisella* MglA–SspA heterodimer evolved as a result of conflicting homodimer contacts in SspA and MglA coupled with the enhancement of favorable contacts in the heterodimer. In particular, the residues in positions corresponding to *F. tularensis* SspA Tyr68/Ile87 appear key in the destabilization of the *Francisella* SspA homodimer while favoring the MglA–SspA heterodimer. These data predict that swapping the alanine or glycine residue found at position 87 in a homodimeric SspA to the *Francisella* SspA isoleucine would abrogate or destabilize this dimerization. As the *F. tularensis* SspA cannot be produced in soluble form in the absence of MglA, we generated a G97I substitution, corresponding to position 87 of *Francisella* SspA (Fig. 2B) in the *E. coli* SspA protein and performed size exclusion chromatography (SEC) experiments. Strikingly, while the wild-type protein was clearly dimeric, *E. coli* SspA(G97I) eluted as a monomer even at concentrations far beyond those expected in cells (Fig. 2D). Thus, these data support the hypothesis that these tyrosine and isoleucine residues are key determinants of the oligomeric state of SspA proteins.

### MglA–SspA interacts directly and specifically with ppGpp

**Figure 3.**
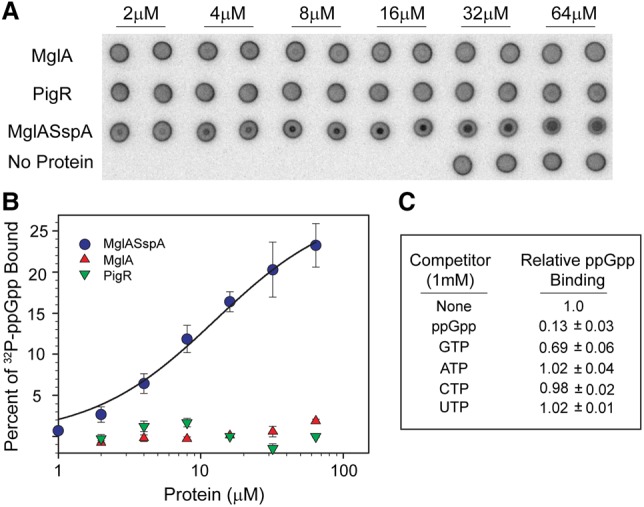
The MglA–SspA heterodimer binds ppGpp. (*A*) Nitrocellulose filter-binding assays from a representative DRaCALA experiment. ^32^P-ppGpp (10 nM) and varying concentrations of purified MglA–SspA, MglA, or PigR (2–64 µM) were used. Filters from samples lacking protein are shown in the *bottom* row (buffer) as a control. (*B*) Plot showing the percentage of total ^32^P-ppGpp counts bound as a function of protein concentration (1–64 μM). Values are averages with standard deviations from multiple independent experiments. For wild-type MglA–SspA, *n* = 7; for MglA or PigR, *n* = 2. (*C*) The effect of unlabeled competitor nucleotides on ^32^P-ppGpp binding to 16 µM MglA–SspA. Values were normalized to that for binding to 16 μM MglA–SspA in the absence of added competitor and are averages from two independent experiments.

Previous work indicated a potential role for ppGpp in promoting contacts between MglA–SspA and PigR, while the interaction between MglA–SspA and RNAP was shown to be independent of ppGpp (Charity et al. 2009). However, it has been unclear whether ppGpp binds to SspA, MglA, or PigR or acts in an indirect manner. Thus, to determine whether ppGpp interacts directly with any of these key FPI regulatory components, we used differential radial capillary action of ligand assays (DRaCALAs) (Roelofs et al. 2011; Ross et al. 2016). Strikingly, DRaCALAs revealed that ppGpp bound to the MglA–SspA complex, while no binding to either PigR or MglA alone was observed (Fig. 3A,B). Binding to *Francisella* SspA alone could not be tested, as, again, soluble protein cannot be produced in the absence of MglA. Mg^2+^ was required for this interaction, and analysis of the data revealed an apparent *K*_d_ of ∼12 µM for the ppGpp–Mg^2+^–(MglA–SspA) interaction (Fig. 3B). This *K*_d_ is in the basal range of ppGpp concentrations measured in bacteria such as *E. coli* (Ryals and Bremer 1982). That MglA–SspA binds ppGpp specifically was supported by competition experiments showing that unlabeled ppGpp effectively competed with ^32^P-ppGpp binding to MglA–SspA, GTP competed weakly, and ATP, CTP, and UTP showed no detectable competition (Fig. 3C).

ppGpp is a ubiquitous bacterial stress signal and second messenger. The finding that this alarmone binds MglA–SspA suggested the possibility that all SspA proteins may bind this molecule. Thus, we used DRaCALAs to analyze whether the *E. coli* SspA homodimer could bind ppGpp. These assays revealed only very weak binding requiring ppGpp concentrations in the high micromolar range (Supplemental Fig. S1). Thus, although the *Francisella* MglA–SspA complex binds ppGpp tightly, the ability to interact with this alarmone is unlikely to be a property shared by all SspA proteins.

### The (MglA–SspA)–ppGpp structure: alarmone binding by a heteromeric SspA complex

**Figure 4.**
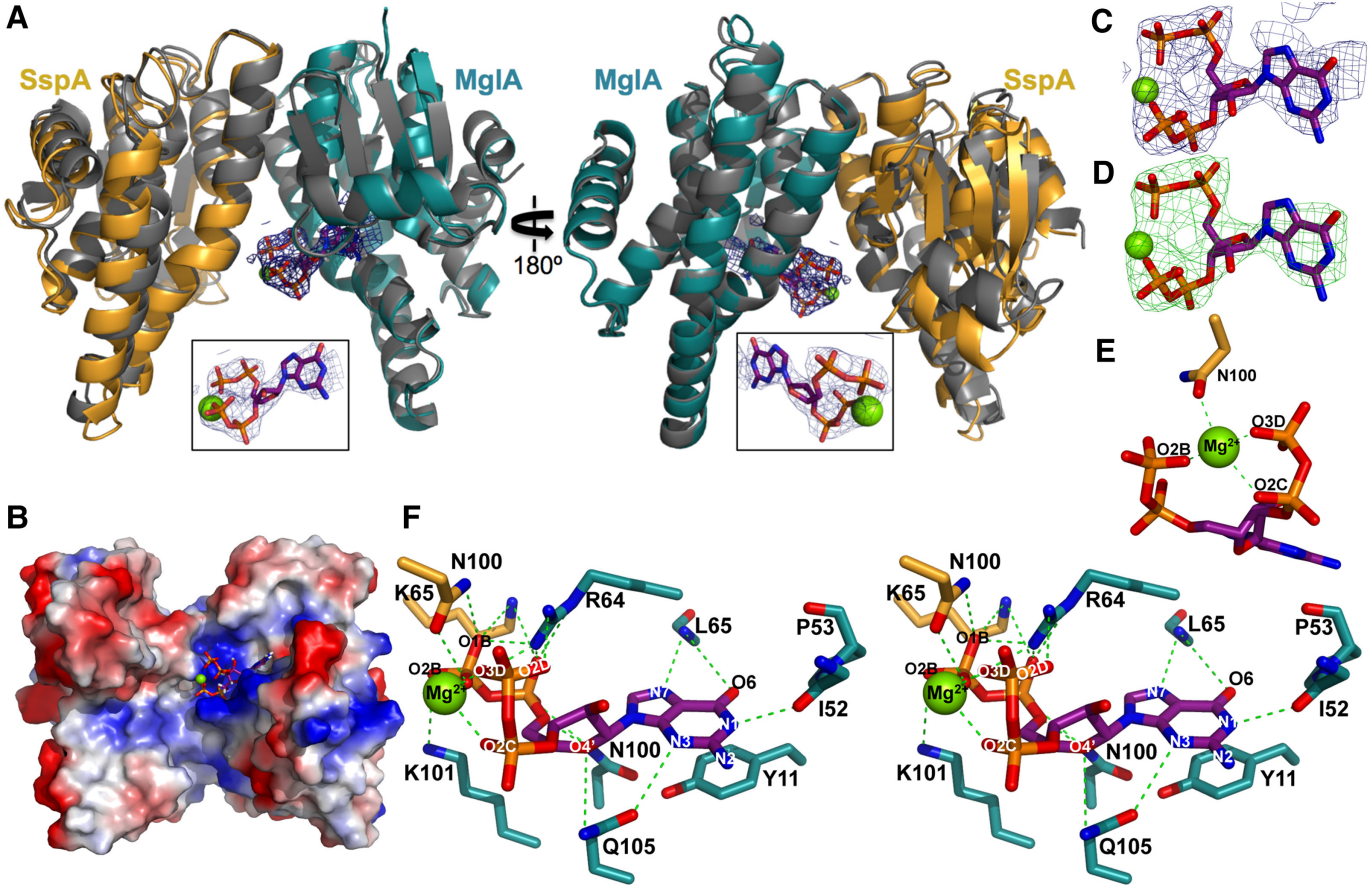
Structure of the (MglA–SspA)–ppGpp complex. (*A*) MglA is shown in teal, and SspA is in yellow. The carbon atoms of ppGpp are purple, and the Mg^2+^ is shown as a green sphere. The overlaid apo–MglA–SspA complex is also shown and colored gray. The *inset* is a view of the bound ppGpp molecule and its 2*F*_o_ − *F*_c_ omit electron density (blue mesh) contoured at 1σ. (*B*) Electrostatic surface of the “open” face of the (MglA–SspA)–ppGpp complex, where blue and red represent electropositive and electronegative surfaces, respectively. (*C*) 2*F*_o_ − *F*_c_ omit electron density of ppGpp (blue mesh) contoured at 1σ. (*D*) *F*_o_ − *F*_c_ omit electron density (green mesh) contoured at 3σ shown for one ppGpp molecule. (*E*) Coordination of Mg^2+^ by ppGpp and SspA residue Asn100. Contacts are shown as green dashes. (*F*) Stereo view of the MglA–SspA-binding site of ppGpp. Hydrogen bonds are shown with dashed green lines.

To understand how a heteromeric SspA/GST family complex can specifically recognize a small molecule ligand, we went on to obtain the structure of the (MglA–SspA)–ppGpp complex. As it was unclear whether ppGpp binding to the complex elicits conformational changes, we carried out de novo crystallization to obtain the (MglA–SspA)–ppGpp structure (Materials and Methods). The structure was solved to 2.8 Å resolution and refined to final *R*_work_/*R*_free_ values of 21.2%/26.5% (Table 1). Like the apo structure, the (MglA–SspA)–ppGpp complex structure contains two MglA–SspA heterodimers in the ASU. In each dimer, clear electron density was observed for a single ppGpp–Mg^2+^ molecule that, strikingly, binds in the open face of the heterodimer with a stoichiometry of one ppGpp to one MglA–SspA dimer (Fig. 4A). Comparison of the apo- and ppGpp-bound MglA–SspA structures showed that the individual subunits and the heterodimer did not undergo any large structural changes upon ppGpp binding (superimposition of the apo- and ppGpp-bound heterodimers gives RMSDs of 0.7–0.8 Å). A previous study suggested that another small molecule involved in signaling, the inorganic polyphosphate hexametaphosphate (hexaMP), binds the MglA–SspA complex (Wrench et al. 2013b). However, we found that hexaMP did not compete with ppGpp binding to MglA–SspA (Supplemental Fig. S2A,B). Moreover, cocrystallization of MglA–SspA with hexaMP revealed no electron density for this small molecule anywhere in the structure (Supplemental Fig. S3).

The majority of the interactions to the ppGpp in the MglA–SspA–ppGpp structure is provided by MglA. Analysis of the electrostatic surface of the ppGpp-binding site revealed that it harbors a large positive patch, and most of these positively charged ppGpp-binding residues are contributed by MglA (Fig. 4B). As evidenced by its low B factors, which are in the range of the well-ordered core of the protein, the individual ppGpp molecules are bound tightly to the complex (Table 1; Fig. 4C,D). Notably, the (MglA–SspA)–ppGpp stoichiometry of one ligand to one heterodimer is distinct from the two GSH ligands to one dimer-binding stoichiometry exhibited by all other GST proteins (Oakley 2011). In the (MglA–SspA)–ppGpp structure, the single ppGpp binds close to the dimer interface, in contrast to GSH, which binds within each monomer of a GST dimer. The fact that the MglA–SspA complex contains a more open face than GST dimers is critical, as this face is where the ppGpp binds. Indeed, modeling shows that GST homodimeric proteins cannot accommodate ppGpp within their narrowed dimer interface (Supplemental Fig. S4).

### MglA–SspA binds ppGpp with high specificity

Structures of effector proteins bound to ppGpp have revealed two main binding modes: one with the alarmone complexed in an elongated state and one with it bound in a ring-like conformation (Steinchen and Bange 2016). The ppGpp in the (MglA–SspA)–ppGpp complex adopts a ring-like conformation, which is stabilized by a bound Mg^2+^ (Fig. 4). Interestingly, the ring-like conformation appears to bind effector proteins with ∼10-fold higher affinity than the elongated state, whereby the ring-like conformer of ppGpp binds with low micromolar affinities, and the elongated state binds with mid to high micromolar affinities (Steinchen and Bange 2016). In accord with this, the affinity of MglA–SspA for ppGpp (∼12 µM) is similar to other proteins that bind ppGpp in the ring-like conformation. Binding of the Mg^2+^ to the phosphates is crucial for stabilizing the bound ring-like structure, which assumes a conformation optimal for docking in the MglA–SspA-binding pocket (Fig. 4E,F). Consistent with a requirement for Mg^2+^, there was little or no binding of ppGpp to MglA–SspA in the absence of this divalent cation.

Clearly, DRaCALAs demonstrated that MglA–SspA binding is highly specific for ppGpp, although, maybe not unexpectedly, GTP at high concentration competes weakly (Fig. 3C). The structure reveals that this is due primarily to specific hydrogen bonds to the guanine base provided by peptide backbone atoms of MglA residues (Fig. 4F). Because the peptide backbone displays little conformational flexibility, it can provide a high level of ligand specificity. The backbone amide group of MglA residue Leu65 and the carbonyl oxygen moiety of MglA residue Ile52 play key roles in guanine base recognition by making hydrogen bonds to the O6 and N7 atoms, respectively. These contacts discriminate against adenine. The only side chain contact to the guanine is from MglA residue Gln105. In addition to discriminating against adenine-containing nucleotides, these interactions preclude binding to pyrimidine nucleotides, which are too small to participate in the aforementioned contacts. Aside from the base-specific interactions with the guanine, additional hydrophilic and hydrophobic contacts anchor the ppGpp molecule into the binding pocket. These contacts include stacking interactions between MglA residue Tyr11 and the guanine base as well as ribose hydroxyl interactions from MglA residues Asn100 and Gln105 (Fig. 4F). The ppGpp pyrophosphate moieties are contacted by the side chains of MglA residues Arg64 and Lys101. Intriguingly, SspA makes only two interactions with the bound ppGpp-Mg^2+^: one from residue Lys65 to the ppGpp phosphate groups and another from residue Asn100, which interacts with the Mg^2+^ ion (Fig. 4E,F).

### Interactions between MglA–SspA and PigR

**Figure 5.**
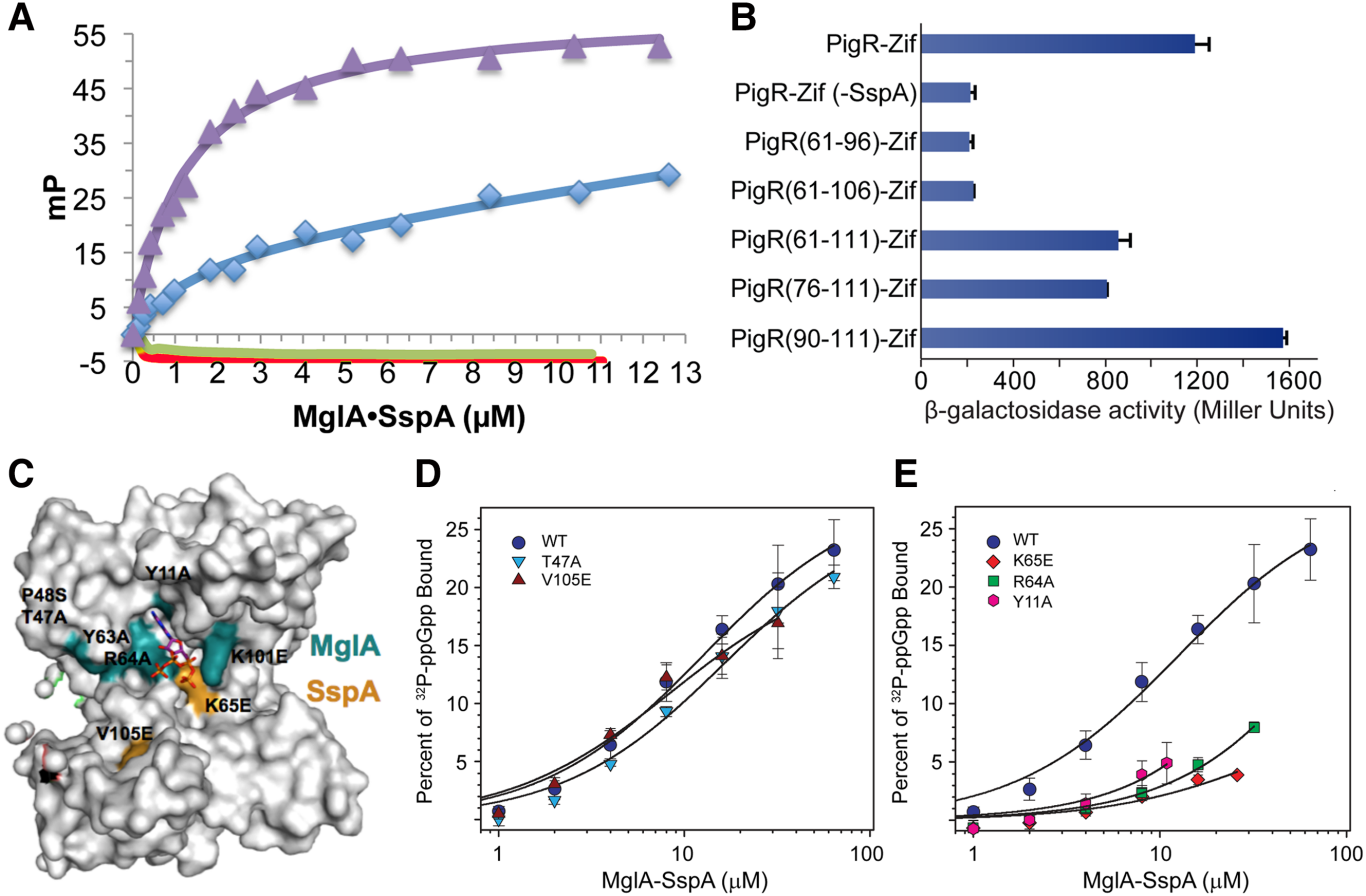
MglA–SspA interacts with the C-terminal tail of PigR. (*A*) Fluorescence polarization experiments examining binding of MglA–SspA to fluoresceinated peptides encompassing N-terminal and C-terminal regions of PigR. Plots show binding isotherms for MglA–SspA binding to PigR(1–34) in the absence of ppGpp (red plot), PigR(1–34) in the presence of ppGpp (green plot), PigR(90–111) in the absence of ppGpp (blue plot), and PigR(90–111) in the presence of ppGpp (purple plot). (*B*) Bridge-hybrid data show that MglA–SspA interacts with PigR, PigR(61–111), PigR(76–111), and PigR(90–111) but not PigR(61–96) or PigR(61–106). (*C*) Mapping of previously identified PigR interaction mutants (colored teal and yellow) onto the (MglA–SspA)–ppGpp structure, shown as a surface. (*D*) (MglA–SspA)–^32^P-ppGpp-binding isotherms examining the interaction of ^32^P-ppGpp with wild-type MglA–SspA, MglA–SspA(V105E), and MglA(T47A)-SspA. (*E*) (MglA–SspA)–^32^P-ppGpp-binding isotherms of the interaction of ^32^P-ppGpp with wild-type MglA–SspA, MglA–SspA(K65E), MglA(Y11A)–SspA, and MglA(R64A)–SspA. Values for MglA–SspA variants are averages with standard deviations from three or four independent DRaCALA experiments.

In addition to the (MglA–SspA)–ppGpp complex, PigR is also essential for FPI activation (Charity et al. 2009). Recent ChIP-seq analyses revealed that MglA–SspA and PigR colocalize to the same promoters (Ramsey et al. 2015). These studies, in addition to bacterial bridge-hybrid assays (Charity et al. 2009; Rohlfing and Dove 2014), suggest that MglA–SspA and PigR interact. In contrast, Brotcke and Monack (2008) provided data arguing against such an interaction. However, none of these studies assessed whether MglA–SspA bound PigR using purified components, which would reveal a direct interaction. The 111-residue PigR protein consists of three main regions: an N-terminal arm (residues 1–34) that is predicted to be disordered, a central MerR-like winged HTH (wHTH) motif (residues 35–89), and a C-terminal region (residues 90–111) that is also predicted to be largely unfolded. We reasoned that the wHTH region, which is typically involved in DNA binding, is unlikely to be used as a protein–protein-binding module and hence we tested the ability of a fluorescently labeled PigR N-terminal peptide, residues 1–34, and a fluorescently labeled C-terminal peptide, residues 90–111, to bind MglA–SspA using a fluorescence polarization-based binding assay (Materials and Methods). Strikingly, whereas the N-terminal peptide did not interact with purified MglA–SspA, the C-terminal PigR peptide KRNVFSRCWINMNLYSVIKAKS showed specific binding (*K*_d_ = 0.95 μM ± 0.50 μM) to the heterodimer but only in the presence of ppGpp. While binding was observed to this peptide in the absence of ppGpp, it was not saturable, indicating nonspecific or very low-affinity binding (Fig. 5A).

To further narrow down the MglA–SspA-binding site in the PigR C-terminal region, we synthesized smaller peptides: a 17mer in which the C-terminal KSKI residues were removed (KRNVFSRCWINMNLYSV) and a peptide that encompassed the last 13 residues of PigR (WINMNLYSVIKAKS). Notably, the latter peptide showed no binding to MglA–SspA in either the presence or absence of ppGpp. However, the 17mer peptide showed robust binding (0.69 μM ± 0.04 μM) (Supplemental Fig. S6). Similar to the fluorescence polarization experiments with the 22mer PigR C-terminal peptide, saturable binding by MglA–SspA to the 17mer was observed only in the presence of ppGpp (Supplemental Fig. S6). Thus, these data indicate that ppGpp binding to MglA–SspA is required to mediate specific and high-affinity binding to the PigR protein, consistent with data showing that ppGpp is key to FPI transcription activation (Charity et al. 2009).

To further probe the interaction of various PigR regions with MglA–SspA, we used the bridge-hybrid that was used previously to interrogate the PigR–(MglA–SspA) interaction (Materials and Methods; Supplemental Fig. S5; Charity et al. 2009; Rohlfing and Dove 2014). This assay was performed in cells grown to mid-log in LB medium, a condition in which ppGpp concentrations are relatively low in *E. coli* (Ryals and Bremer 1982) but are apparently sufficient to support complex formation. Using this cell-based system, we analyzed the ability of PigR residues 1–111 (the full-length protein), 61–96, 61–106, 61–111, 76–111, and 90–111 to interact with MglA–SspA. Interactions were detected between MglA–SspA and PigR residues 1–111, 61–111, 76–111, and 90–111 but not with PigR residues 61–96 or 61–106 (Fig. 5B). Thus, these experiments demonstrate that the PigR C-terminal region binds MglA–SspA, consistent with our fluorescence polarization analyses.

### Probing the MglA–SspA ppGpp-binding mechanism

Previous bridge-hybrid results showed that MglA mutants Y11A, T47A, P48S, Y63A, R64A, and K101E and SspA mutants K65E and V105E displayed decreased interaction with PigR (Rohlfing and Dove 2014). Notably, our structure shows that these residues are either proximal to the ppGpp-binding site or directly involved in ppGpp binding (Fig. 5C). These findings are interesting given our data showing that the presence of ppGpp affects PigR binding to MglA–SspA. Thus, we next used DRaCALAs to assess the ability of the mutant heterodimers MglA(Y11A)–SspA, MglA(R64A)–SspA, MglA–SspA(K65E), MglA(T47A)–SspA, and MglA–SspA(V105E) to bind ppGpp. Notably, substitutions to residues that contact ppGpp in the structure—MglA(Y11A)–SspA, MglA(R64A)–SspA, and MglA–SspA(K65E)—resulted in severely impaired ppGpp binding (Fig. 5E), while mutations to non-ppGpp-coordinating residues MglA(T47A)–SspA and MglA–SspA(V105E) resulted in near wild-type ppGpp binding (less than twofold reduced) (Fig. 5D). Thus, these results support the crystallographically determined (MglA–SspA)–ppGpp-binding mode and also the hypothesis, revealed by our fluorescence polarization studies, that ppGpp binding to MglA–SspA is important in mediating the interaction of the complex with PigR.

## Discussion

The ability of *F. tularensis* to adapt to diverse environmental conditions and infect multiple hosts has led to its evolution as one of the most infectious bacteria known. Previous studies suggest that the alarmone ppGpp, which is produced during stress, is the signaling molecule that triggers the *F. tularensis* virulence program (Charity et al. 2009). An unusual combination of transcription factors comprised of SspA, MglA, and PigR mediates activation of the FPI and hence is essential for *Francisella* virulence. Here, we dissected the molecular mechanisms controlling this system and revealed a direct connection between these virulence regulators and ppGpp.

Unlike other bacterial SspA proteins, *Francisella* SspA does not homodimerize but rather forms a complex with MglA. Our structures of MglA–SspA complexes revealed that the proteins form a specific heterodimer that harbors an open and positively charged cavity (Fig. 4B). The structure also revealed the basis for heterodimer preference in which residues that would be proximal in homodimers of *Francisella* SspA and MglA would lead to steric clash or electrostatic repulsion. In particular, positions corresponding to Tyr68 and Ile87 in *Francisella* SspA were shown to be critical for selective dimerization. The bulky side chains of these residues would collide in any *Francisella* SspA homodimer, but the Ile87 residue is replaced by smaller residues, either alanine or glycine, in SspAs that preclude clash and hence are able to form homodimers. In *Francisella* MglA, both residues are alanines, thereby also averting a collision. Unlike SspA, MglA can form nonphysiological homodimers at very high concentrations (Cuthbert et al. 2015). Inspection of the MglA dimer interface reveals the proximal placement of basic residues, which is unfavorable when compared with the MglA–SspA heterodimer, in which one of these basic residues is replaced by an acidic residue. The preferential heterodimerization of MglA with SspA is somewhat analogous to that observed between the eukaryotic basic region-leucine zipper transcription factors Fos and Jun (O'Shea et al. 1992). Similar to MglA, Jun can homodimerize but prefers to form heterodimers with Fos. Fos, like *Francisella* SspA, does not form stable homodimers. In the case of Fos dimerization, this is because of the electrostatic repulsion between acidic residues in the e and g′ positions of the two interacting coiled-coil helices. In contrast, Jun harbors basic residues at the key positions that form favorable electrostatic contacts with Fos.

The pivotal role that MglA–SspA plays in *Francisella* virulence was revealed by our studies showing that this complex binds directly to the stress signal ppGpp. To ascertain how this small molecule is bound by a heterodimeric GST-like complex, we obtained the structure of the (MglA–SspA)–ppGpp complex. The structure reveals a novel mode of ligand binding for a GST protein. Specifically, a single ppGpp was bound to the heterodimer, with most of the ppGpp contacts provided by MglA. This interaction shows why MglA–SspA, unlike GST proteins, harbors an open cavity on one of its faces, as this cavity allows the specific binding of ppGpp. Intriguingly, ppGpp binding to MglA–SspA does not result in a large conformational change; hence, how it transmits the signal of stress through this complex was unclear. Insight into this quandary was revealed by our studies that showed that PigR binds directly to MglA–SspA and that ppGpp must be bound to the MglA–SspA complex to permit a high-affinity interaction with PigR. Because the ppGpp bound by MglA–SspA is exposed in the structure, it is likely that PigR interacts directly with the alarmone as well as MglA–SspA residues. ppGpp binds specifically to a variety of protein targets (Kanjee et al. 2012). In *E. coli* RNAP (Ross et al. 2013, 2016; Zuo et al. 2013), ppGpp binds to two sites: site 1, which is at the interface of the ω and β′ subunits, and site 2, which is a pocket formed by the interaction of β' and the transcription factor DksA. In *E. coli*, hundreds of promoters are regulated negatively and hundreds more are regulated positively by ppGpp (Durfee et al. 2008; Traxler et al. 2008). Binding of ppGpp to either site 1 or site 2 can inhibit transcription, but only binding of ppGpp to site 2 can stimulate transcription (Ross et al. 2016). Although both sites are generally well conserved in proteobacteria (Ross et al. 2013, 2016), bioinformatic analyses suggest that *F. tularensis* is an exception. The amino acid residues that form site 1 are present in *F. tularensis*, and ppGpp appears to cross-link to a high-molecular-weight protein band, consistent with binding to RNAP (W Ross, SL Dove, and RL Gourse, unpubl.), but, thus far, we have been unable to identify a gene in the *F. tularensis* genome sequence that codes for a DksA homolog. Since DksA is required for formation of ppGpp site 2 and since site 2 is necessary and sufficient for activation by ppGpp in other γ-proteobacteria, it appears that (MglA–SspA)–PigR functionally replaces site 2 for transcription activation by ppGpp in *F. tularensis*. Further studies will be required to test this hypothesis and evaluate its regulatory significance.

The predicted wHTH of PigR places it in the MerR family of transcription regulators. However, PigR is distinct from canonical MerR members in that its wHTH motif is not at its very N terminus and it is not predicted to contain the coiled-coil domain that is characteristic of canonical MerR proteins. Thus, PigR might be better placed within the recently characterized TnrA/GlnR subfamily of MerR proteins (Schumacher et al. 2015). TnrA/GlnR proteins do not contain coiled coils and are monomers in their apo form. These proteins dimerize upon DNA binding through residues in their N-terminal regions (Schumacher et al. 2015). TnrA and GlnR possess unstructured C-terminal regions, which in both proteins are responsible for contacts with glutamine synthetase (GS). Binding of the C-terminal tail of TnrA to GS deactivates TnrA as it pulls apart the weak DNA-binding dimer. In contrast, GS binding to the GlnR C-terminal tail disrupts an autoinhibitory interaction of this tail with the wHTH, thus activating GlnR to bind DNA (Schumacher et al. 2015). In a similar manner, MglA–SspA binding to the PigR C-terminal tail may alter its ability to bind DNA. However, unlike TnrA and GlnR, PigR may bind DNA as a monomer, since PigR appears to bind a 7-bp nonpalindromic site (PRE), whereas dimers of TnrA and GlnR bind 18-bp palindromic sites (Ramsey et al. 2015; Schumacher et al. 2015).

**Figure 6.**
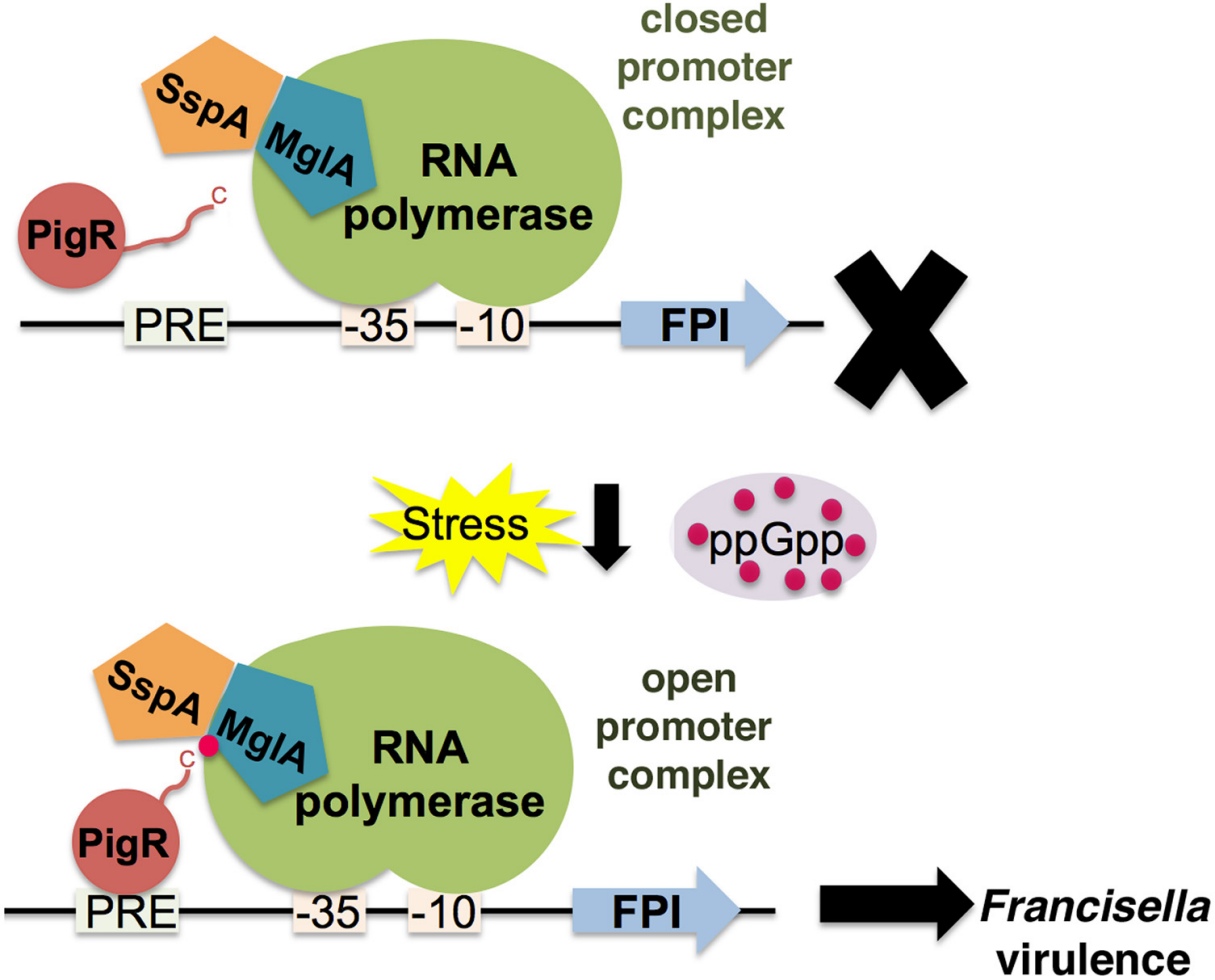
Model of the molecular virulence circuitry of *F. tularensis*. In the model, MglA and SspA form an obligate heterodimer. ppGpp (dark-pink circles), which is produced upon infection/stress, binds specifically to the MglA–SspA complex. In the absence of ppGpp, PigR associates weakly with MglA–SspA. ppGpp binding effects high-affinity binding by PigR to the MglA–SspA complex. Formation of the MglA–SspA–ppGpp–PigR complex enables DNA binding by PigR to PRE-containing promoters. The MglA–SspA-bound RNAP is now bound stably, thus facilitating open promoter complex formation and FPI transcription.

Thus, our studies show that *Francisella* has evolved a unique set of regulators to control virulence. Furthermore, our structural, biochemical, and cellular analyses suggest a model for the assembly of the *Francisella* virulence machinery (Fig. 6). According to this model, an increase in ppGpp concentration due to the multiple stresses of infection would lead to binding of this alarmone to the MglA–SspA pocket. Importantly, formation of this complex permits a high-affinity interaction with PigR. Previous studies showed that PigR is not found bound to DNA in the absence of MglA–SspA (Ramsey et al. 2015). Thus, the generation of the MglA–SspA–ppGpp–PigR complex likely facilitates the interaction of PigR with a nearby PRE in promoters that are activated by this complex, such as the FPI. As RNAP is bound on the “opposite” face of the MglA–SspA complex, RNAP would be stabilized at the PRE-containing promoter, allowing polymerase to transit from a closed to an open promoter complex, thereby leading to FPI transcription. Last and notably, the data described here have also illuminated surfaces unique to *Francisella* to exploit in the rational design of specific *F. tularensis* anti-virulence chemotherapeutics.

## Materials and methods

### Expression and purification of PigR, MglA–SspA, and MglA

A *his_6_-sspA-(his_6_-mbp-mglA)* coexpression system was generated by cotransforming plasmids encoding *F. tularensis sspA* (which was cloned into the pMCSG21 vector using ligation-independent cloning) and the *his_6_-mpb-mglA* fusion (cloned into pET28A), into C41(DE3) cells (Cuthbert et al. 2015). For protein expression, the vector-containing C41(DE3) cells were grown to an OD_600_ of 0.5 at 37°C and induced by addition of 0.5 mM isopropyl β-D-1-thiogalactopyranoside (IPTG) overnight at 15°C. Protein purification was performed as described previously (Cuthbert et al. 2015). The *pigR* gene was generated and codon-optimized for *E. coli* expression by Genscript Corporation. The gene was subcloned via ligation-independent cloning into the 2Bc-T vector. This construct generated a PigR protein with a C-terminal his_6_ tag. For protein expression, the *pigR* construct-containing cells were grown to an OD_600_ of 0.5 at 37°C and induced with 0.5 mM IPTG for 3.5 h at 37°C. The cells were lysed with 7.5% N-lauroylsarcosine (sarkosyl) in buffer A (25 mM Tris at pH 7.5, 300 mM NaCl, 5% glycerol). The PigR protein was purified by Ni-NTA affinity chromatography using buffer A supplemented with 0.5% sarkosyl and increasing amounts of imidazole. The *E. coli sspA* gene, optimized for *E. coli* expression, was purchased from Genscript and was subcloned into pET15b such that it encoded an N-terminal his_6_ tag. The *E. coli sspA-*containing vector was transformed into C41(DE3) cells, and protein expression was induced by the addition of 0.5 mM IPTG to cells grown to an OD_600_ of 0.5 for 4 h at 37°C. The expressed protein was purified via Ni-NTA chromatography in buffer A followed by SEC.

### Structure determination of apo- and ppGpp-bound MglA–SspA complexes

The wild-type MglA–SspA complex did not produce data-quality crystals. Hence, mutagenesis was used to generate small truncations in the N-terminal and C-terminal regions of both proteins. Ultimately, an N-terminally truncated SspA in which two residues were removed and a C-terminally truncated MglA in which four residues were removed produced data-quality crystals of the heterodimer. Crystals were grown using the hanging drop vapor diffusion method. Specifically, the complex at 26 mg/mL was mixed 2:1 with a crystallization solution consisting of 0.2 M L-proline, 0.1 M HEPES (pH 7.5), and 24% PEG 1500. The crystals were cryoprotected by dipping them for several seconds in a drop containing the crystallization solution supplemented with 6% glycerol. X-ray intensity data were collected to 2.65 Å resolution at the Advanced Light Source (ALS) beamline 5.0.1. The data were processed and scaled using HKL2000 (Otwinowski and Minor 1997). Phaser in CCP4 was used to solve the structure by molecular replacement using the subunits from the *F. tularensis* MglA (Protein Data Bank [PDB] ID 4PUR) and *Yersinia pestis* SspA (PDB ID 1YY7) structures as search models (Hansen et al. 2005; Winn et al. 2011; Cuthbert et al. 2015). Two clear solutions were obtained for each; the crystallographic ASU contained two MglA–SspA dimers. Coot was used to replace the *Y. pestis* SspA sequence with that of the *F. tularensis* SspA. After multiple rounds of rebuilding, refinement in Phenix, and validation using MolProbity, the model converged to *R*_work_/*R*_free_ values of 20.5%/25.9% (Table 1; Emsley and Cowtan 2004; Adams et al. 2010).

The (MglA–SspA)–ppGpp complex was crystallized de novo using hanging drop vapor diffusion and a solution of 8 mg/mL MglA–SspA, 10 mM MgCl_2_, and 3 mM ppGpp and a crystallization solution consisting of 0.1 M Tris (pH 8.5) and 22% PEG 3350. The crystals were cryopreserved by dipping them in the crystallization solution supplemented with 6% glycerol for several seconds. X-ray intensity data were collected on ALS beamline 5.0.2 to 2.80 Å resolution. Data were indexed and integrated using Mosflm and scaled using Scala in CCP4 (Winn et al. 2011). Molecular replacement was performed with subunits from the apo MglA–SspA crystal structure. Model building and refinement were carried out using Coot and Phenix, respectively. The final model had *R*_work_/*R*_free_ values of 21.2%/26.5% (Table 1). Crystals of MglA–SspA in the presence of 3 mM hexaMP were obtained (after extensive screening) that were isomorphous with apo MglA–SspA crystals. X-ray intensity data for one of the crystals were collected to 3.28 Å resolution with R-AXIS HTC imaging plates mounted on a Rigaku FRE+ DW Superbright rotating anode generator using Cu Kα radiation. The data were processed using Mosflm. MolRep was used to obtain a molecular replacement solution using the apo MglA–SspA dimer structure as a search model. As found for the apo- and ppGpp-bound structures, there were two MglA–SspA dimers in the ASU. The structure was refined using Phenix to *R*_work_/*R*_free_ values of 23%/28% and revealed no density for hexaMP (Supplemental Fig. S3).

### SEC studies

For SEC analysis of wild-type *E. coli* SspA and *E. coli* SspA(G97I), 5 mg of each purified protein was injected separately onto a Superdex S75 column pre-equilibrated with 20 mM Tris (pH 7.5), 200 mM NaCl, 10% glycerol, and 1 mM β-mercaptoethanol (BME). The molecular weights of the samples in these experiments were calculated from a standard curve generated from the elution volumes of protein standards, aprotin, cytochrome C, carbonic anhydrase, and albumin. Elution volumes were determined by peak integration with PrimeView software.

### ^32^P-ppGpp-binding assay (DRaCALAs)

^32^P-ppGpp binding to purified proteins was measured using DRaCALAs (Roelofs et al. 2011). For these assays, ^32^P-ppGpp was synthesized as described previously (Ross et al. 2013, 2016). Purified ^32^P-ppGpp contained no other labeled compounds and comigrated on an analytical TLC plate with unlabeled ppGpp purchased from TriLink Biotechnologies (imaged by UV shadowing). Fifteen-microliter binding reactions were carried out for 10 min at 22°C with ∼10 nM ^32^P-ppGpp using a range of protein concentrations. The reaction buffer consisted of 20 mM Tris-Cl (pH 7.5), 10 mM MgCl_2_, 100 mM NaCl, and 0.5 mM BME. Competition experiments contained 1 mM unlabeled competitor nucleotides (ppGpp, GTP, ATP, CTP, or UTP). For the assays, 4-µL aliquots were spotted slowly onto dry nitrocellulose filter discs, and dried filters were quantified by phosphorimaging. Protein-bound counts were determined for each filter by correction of counts in the central spot for background of unbound ^32^P-ppGpp and expressed as the percentage of total counts in the entire spot (Roelofs et al. 2011). Radioactivity in the darker outer ring at the edge of the entire spot was not included when determining background correction values but was included in total counts. Duplicate filters were spotted from each reaction, and the values shown represent averages with standard deviations determined from multiple independent experiments. Graphs were created in SigmaPlot with data fit to *y* = Bmax × *X*/(Kd + *X*).

### Fluorescence polarization binding experiments

Fluorescence polarization experiments were performed with fluoresceinated PigR peptides and unlabeled MglA–SspA. For the N-terminal peptide MANQYSGNFEQIVKNRFKCSAREILLKCQ REGLK, the fluorescent tag was added at the C-terminal residue. The C-terminal peptides KRNVFSRCWINMNLYSVIKAKS, KRNVFSRCWINMNLYSV, and WINMNLYSVIKAKS were synthesized with N-terminal fluorescent tags. For fluorescence polarization analyses, the peptides were solubilized in 10 mM sodium cacodylate. Fluorescence polarization experiments were performed in a buffer composed of 20 mM Tris (pH 7.5), 100 mM NaCl, 10% glycerol, and 10 mM MgCl_2_ in the presence or absence of 0.5 mM ppGpp. Data were plotted and fit with Logger Pro 3.8.6.

### Bacterial strains and plasmids

The *E. coli* reporter strain KDZif1ΔZ used for the bridge-hybrid assays and the plasmids pCL, pCL-SspA, pBR-MglA-ω, and pACTR-AP-Zif have been described previously (Vallet-Gely et al. 2005; Charity et al. 2009; Rohlfing and Dove 2014). Plasmid pACTR-PigRN-Zif specifies PigR residues 1–111 fused to Zif, and is similar to plasmid pACTR-PigR-Zif used previously (Charity et al. 2009; Rohlfing and Dove 2014) except the native NdeI site within *pigR* was removed. The plasmid was created by cloning DNA specifying PigR (residues 1–111) but lacking the internal NdeI site into NdeI–NotI-digested pACTR-AP-Zif. Plasmids pACTR-PigR(61–111)-Zif, pACTR-PigR(76–111)-Zif, pACTR-PigR(90–111)-Zif, pACTR-PigR(61–96)-Zif, and pACTR-PigR(61–106)-Zif specified a methionine residue followed by the indicated residues of PigR fused to Zif. These plasmids were made by cloning the appropriate NdeI–NotI-digested PCR products (lacking any NdeI site native to *pigR*) into NdeI–NotI-digested pACTR-AP-Zif. All pACTR-PigR-Zif plasmids directed the synthesis of the specified PigR-Zif fusion protein under the control of the *lac*UV5 promoter.

### Bridge-hybrid assays

For the bridge-hybrid assays, cells were grown in LB supplemented with 100 μg/mL carbenicillin, 10 μg/mL tetracycline, 100 μg/mL spectinomycin, and 50 μM IPTG. β-Galactosidase assays were performed as described previously (Rohlfing and Dove 2014) in triplicate on two separate occasions, and a representative data set is shown. Error bars indicate standard deviation.

### Accession numbers

The MglA–SspA, (MglA–SspA)–ppGpp, and MglA–SspA complexes crystallized in the presence of hexaMP (polyP) coordinates and structure factors have been deposited in the Protein Data Bank under accession codes 5U56, 5U51, and 6ALX, respectively.

## Supplementary Material

Supplemental Material
