# Supplementary material for: Dissection of the molecular circuitry controlling virulence in Francisella tularensis
Source: Genes Dev. 2017 Aug 1;31(15):1549–60. doi: 10.1101/gad.303701.117 (PMC5630020; doi:10.1101/gad.303701.117)
Supplement: Supplemental Material [file supp_31_15_1549__index.html]

Dissection of the molecular circuitry controlling virulence in Francisella tularensis — Supplemental Material 

# Dissection of the molecular circuitry controlling virulence in *Francisella tularensis*

## Supplemental Material

- Supplemental\_Material.pdf
