# Supplementary material for: Dissection of the molecular circuitry controlling virulence in Francisella tularensis
Source: Genes Dev. 2017 Aug 1;31(15):1549–60. doi: 10.1101/gad.303701.117 (PMC5630020; doi:10.1101/gad.303701.117)
Supplement: Supplemental Material [file supp_gad.303701.117_Supplemental_Material.pdf]

SUPPLEMENTAL MATERIAL

Dissection of the molecular circuitry controlling virulence in *Francisella tularensis*

Authors:
Bonnie J. Cuthbert[1], Wilma Ross[2], Amy E. Rohlfing[3,4], Simon L. Dove[3], Richard L. Gourse[2], Richard G. Brennan[1*], Maria A. Schumacher[1*]

Author affiliations:
[1]Department of Biochemistry, Duke University School of Medicine, Durham, North Carolina 27710, USA
[2]Department of Bacteriology, University of Wisconsin-Madison, 1550 Linden Drive, Madison, WI 53706, USA
[3]Division of Infectious Diseases, Boston Children's Hospital, Harvard Medical School, Boston, Massachusetts, USA

Author notes:
Present address
[4]Department of Molecular Biology and Microbiology, Tufts Medical Center, Boston, Massachusetts, USA

*Corresponding authors:
Maria A. Schumacher
Maria.schumacher@duke.edu
(919)-684-9468
Room 243A Nanaline Duke Bldg, 307 Research Dr., Box 3711, Durham, NC 27710

Richard G. Brennan
Richard.brennan@duke.edu
(919)-684-9471
Room 242A Nanaline Duke Bldg, 307 Research Dr., Box 3711, Durham, NC 27710

Contents of Supplemental Material

Figures S1 to S6

Figure S1.

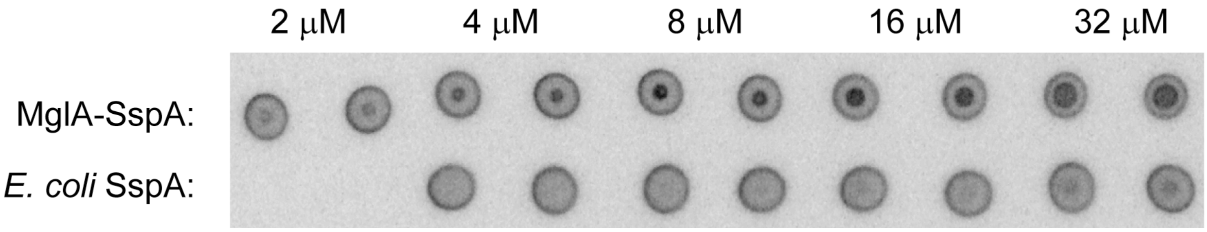

Figure S1. DRaCALA analyzing ^{32}P-ppGpp binding to *F. tularensis* MglA-SspA and *E. coli* homodimeric SspA. Notably, *E. coli* SspA showed essentially no binding even at high μM concentrations of protein.

Figure S2

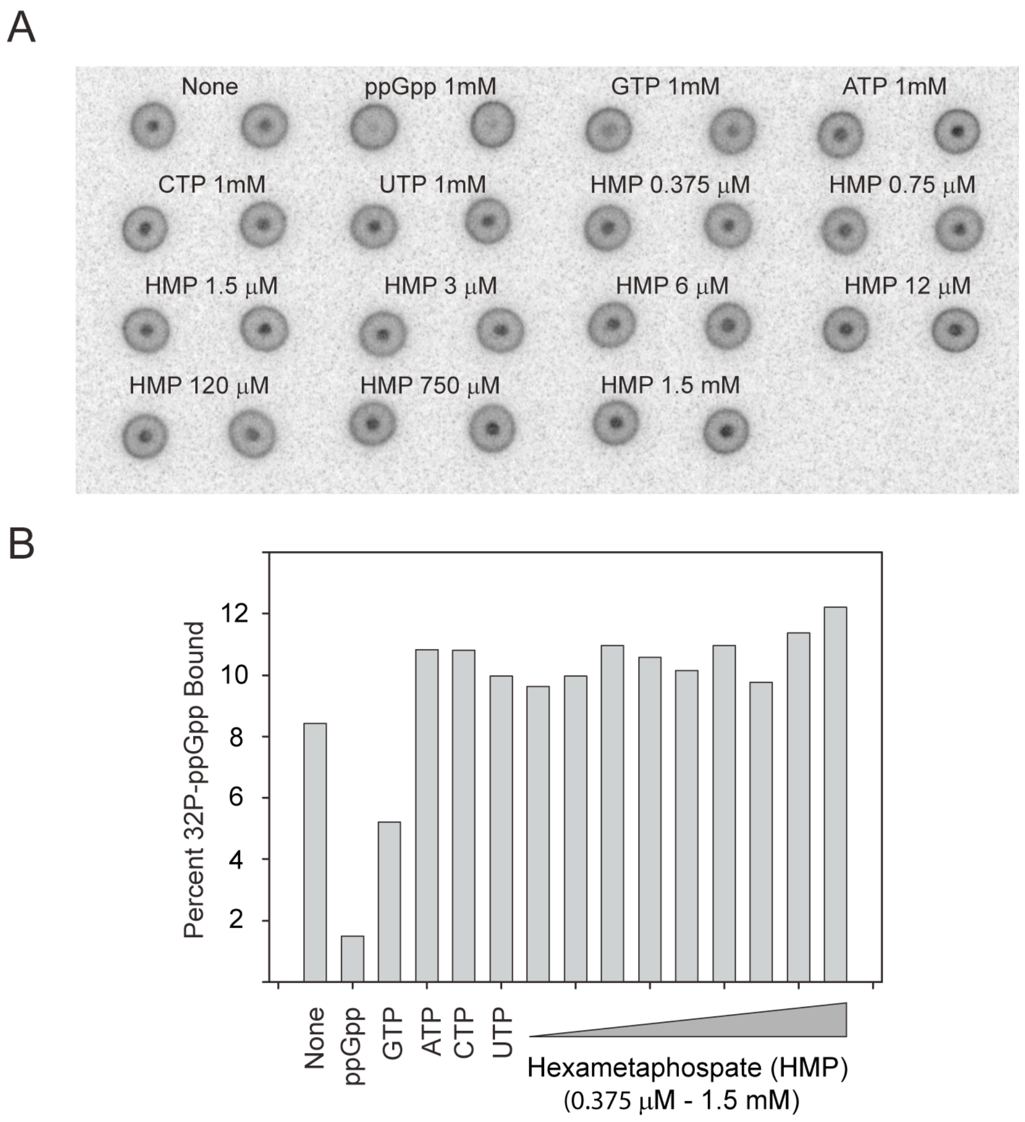

Figure S2. Poly-phosphate (hexaMP) does not compete with ppGpp for MglA-SspA binding. **A**) Competition experiment analyzing the ability of a range of hexametaphosphate (hexaMP) concentrations to compete with ppGpp for MglA-SspA binding. Shown also are controls, ppGpp, GTP, ATP, UTP and CTP. **B**) Quantification of the competition experiment shown in A.

Figure S3

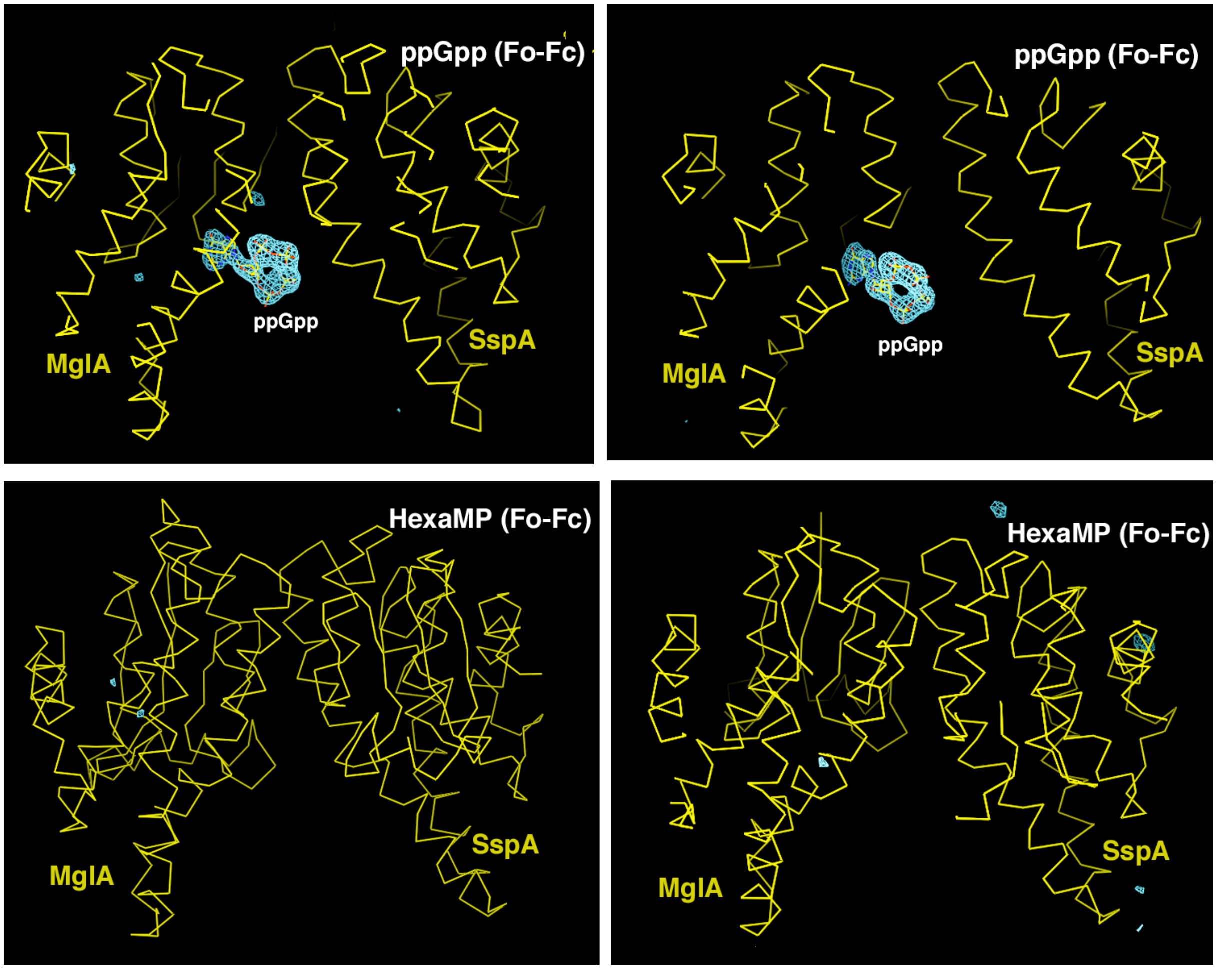

Figure S3. Comparison of Fo-Fc maps from data obtained of crystals of the MglA-SspA complex obtained in the presence of ppGpp (top two panels) (Materials and Methods) or hexaMP (3 mM) (bottom two panels). The maps cover both MglA-SspA subunits in the ASU (each panel) and were contoured to 3.7 σ around the entire dimer in each case. Notably, clear density for ppGpp is observed in each MglA-SspA dimer in the MglA-SspA complex crystallized in the presence of ppGpp while no density for hexaMP is observed for MglA-SspA cocrystallized with hexaMP in either the ppGpp pocket nor any other part of the structure.

Figure S4

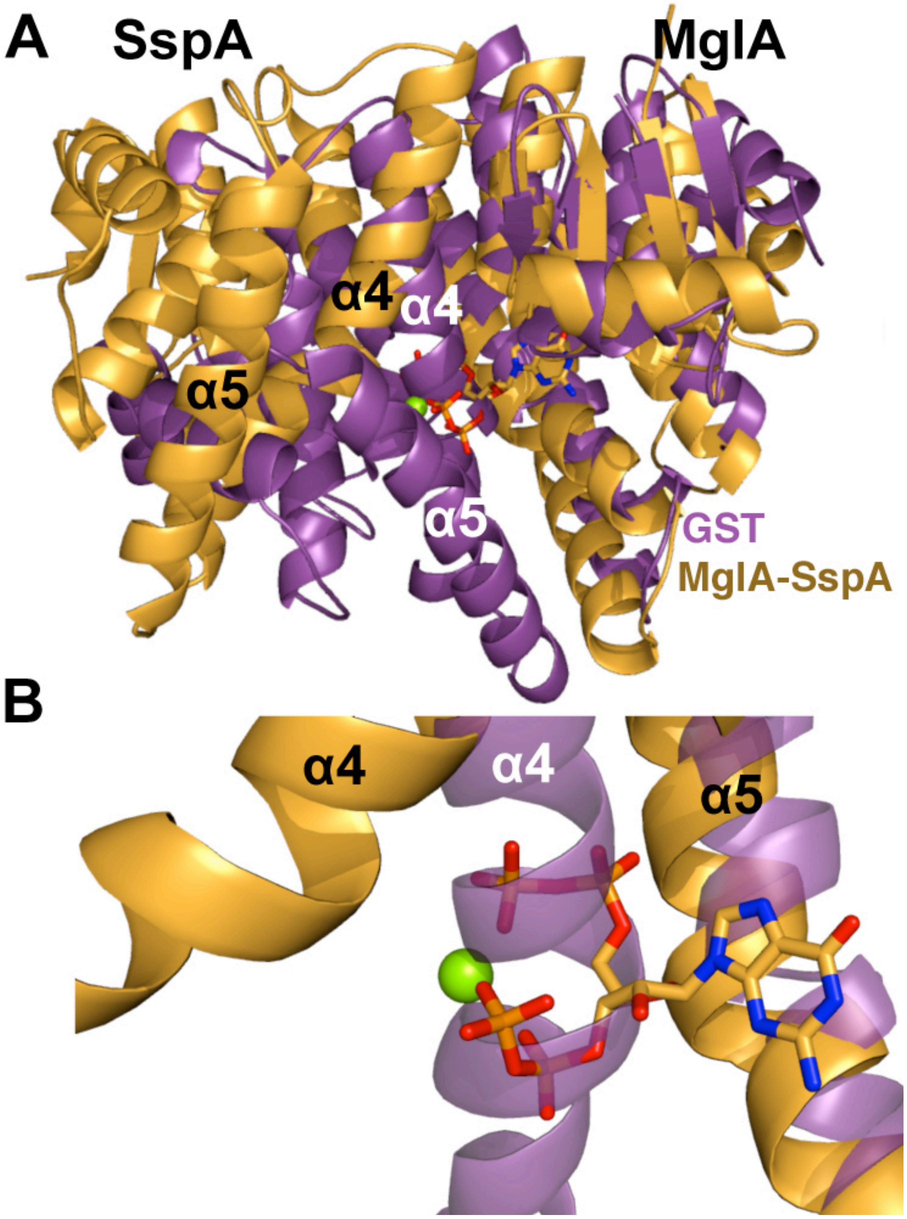

Figure S4. The open face of MglA-SspA distinguishes it from GST dimers and is used to bind ppGpp. **A**) Overlay of one subunit of a GST dimer (purple) onto the MglA subunit of the (MglA-SspA)-ppGpp complex (dark yellow). GST homodimers are more closed and cannot bind ppGpp as clash would result. **B**) The ppGpp binding site clashes with GST α4 helix, but is accommodated by the open face of MglA-SspA.

Figure S5

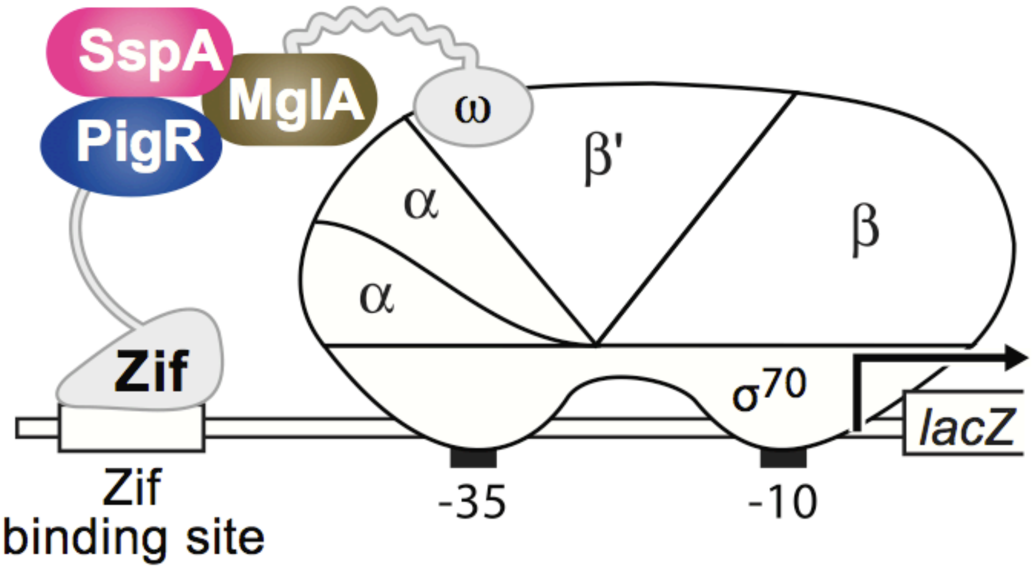

Figure S5. Schematic of the *E. coli* bridge-hybrid assay employed to show interaction between MglA•SspA and PigR. Interaction between MglA-ω•SspA and PigR-Zif induces transcription of *lacZ*.

Figure S6

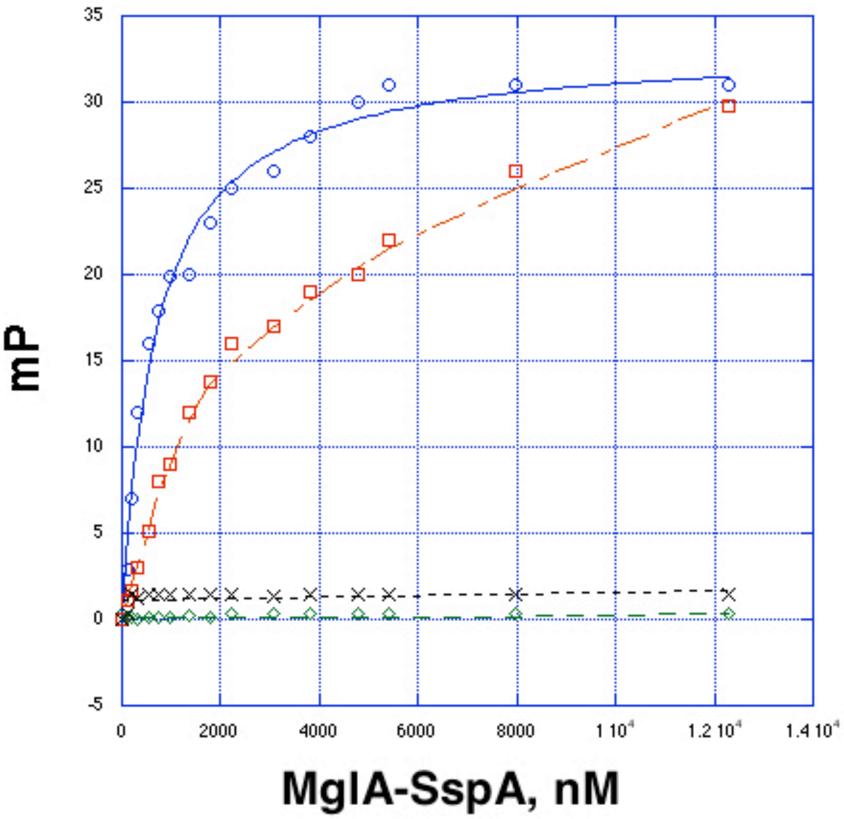

Figure S6. FP binding isotherm of MglA-SspA to the C-terminal 17mer PigR peptide (KRNVFSRCWINMNLYSV) in the presence (blue) and absence (red) of ppGpp. In the absence of ppGpp binding is nonspecific and non-saturable while in the presence of ppGpp a K_d of 0.69 ± 0.04 μM is obtained. Also shown are the binding isotherms for MglA-SspA binding to the 14mer PigR C-terminal peptide (WINMNLYSVIKAKS) in the presence (black) and absence (green) of ppGpp. No binding was observed in either case.
